# Microbial Influence on Carbon Storage and Trace Element Speciation in Restored and Natural Mangrove Sediments: A Synthesis of the Current Understanding

**DOI:** 10.3390/microorganisms14081662

**Published:** 2026-07-30

**Authors:** Mohammad Mazbah Uddin, Tariqul Islam, M. M. Abdullah Al Mamun, Md. Akramul Islam, Kang Mei, Chengfeng Xue, Yining Chen

**Affiliations:** 1 Second Institute of Oceanography, Ministry of Natural Resources, Hangzhou 310012, China; mazbahuddin@sio.org.cn (M.M.U.); cfxue@sio.org.cn (C.X.); 2 Department of Agricultural Construction and Environmental Engineering, Sylhet Agricultural University, Sylhet 3100, Bangladesh; tariqul.acee@sau.ac.bd; 3 Institute of Forestry and Environmental Sciences, University of Chittagong, Chittagong 4331, Bangladesh; mamun@cu.ac.bd; 4 State Key Laboratory of Lake and Watershed Science for Water Security, Nanjing Institute of Geography and Limnology, Chinese Academy of Sciences, Nanjing 211135, China; akramkukhulna@gmail.com; 5 Jiangsu Institute of Marine Resources Development, Jiangsu Key Laboratory of Marine Bioresources and Environment, Jiangsu Ocean University, Lianyungang 222005, China; kangmei@jou.edu.cn

**Keywords:** microbial diversity, carbon storage, trace elements, accumulation, speciation, mangrove ecosystems

## Abstract

Mangrove microorganisms play a fundamental role in regulating sediment biogeochemical processes, particularly carbon storage and trace element cycling. Although numerous studies have examined microbial roles in individual processes, an integrated understanding of how microbial communities simultaneously regulate carbon storage and trace element dynamics in natural and restored mangrove ecosystems remains limited. This review synthesizes the global status of mangrove microbial research and the influence of microbes on carbon storage, trace element accumulation, and speciation in natural and restored mangrove ecosystems, while identifying emerging research trends and knowledge gaps. Our investigation revealed that research on the influence of microbes on carbon storage in mangrove sediments is increasing globally, with considerably increasing trends after 2017. However, less research has been reported on microbial trace metal interrelations than on carbon storage relationships, suggesting that there is limited focus from researchers on this topic. The available evidence indicates that sulfate reduction, microbial extracellular polymeric substances (EPS), microbial necromass formation, redox-driven biogeochemical coupling, and microbially mediated mineral transformations are the principal mechanisms promoting long-term carbon stabilization in mangrove sediments. Therefore, several studies have also suggested that microbial diversity regulates trace element accumulation and speciation through different pathways, such as redox transformation, bioadsorption, EPS-mediated binding or aggregation, biomineralization, and sulfide precipitation, in mangrove sediment. The principal conceptual contribution of this review is the development of an integrated framework demonstrating that microbial processes act as a central biogeochemical bridge connecting carbon storage and trace element cycling, rather than regulating these functions independently. Finally, we identify critical challenges and research priorities, including functional gene characterization, integrated metal–microbe–plant interactions, multi-omics approaches, long-term monitoring, and global meta-analyses, to improve mechanistic understanding and support evidence-based mangrove restoration and blue carbon management.

## 1. Introduction

Mangrove ecosystems play a vital role in protecting coastlines from erosion, while providing essential habitats for plants, animals, and microbial communities [1]. In addition to their ecological significance, mangroves deliver important ecological services including carbon sequestration, trace element cycling, and nutrient regulation [2,3,4]. Recently, carbon sinks by mangrove forests have been highlighted globally because they are vital reservoirs of carbon stocks. Recent estimates indicate that global mangrove organic carbon stocks are approximately 1.93 pg, lower than previous assessments [5]. However, the whole mangrove ecosystem’s carbon stock (dead biomass, organic and inorganic) is estimated to range from 3.7 to 6.2 pg [5]. Furthermore, mangrove forests contribute approximately 4.5 Mg C ha^−1^ yr^−1^ to global net ecosystem productivity, corresponding to an annual carbon sequestration of about 66.6 Tg C [6].

Microbial communities are fundamental to the functioning of mangrove ecosystems, contributing to ecosystem health, stability, organic matter decomposition, and the movement of nutrients or trace elements [7,8,9]. Sediment microorganisms are important for the biogeochemical cycle of nutrients, changes in the redox state, and the bioavailability of trace elements in sediment through their metabolic activity [10]. Increasing evidence indicates that microbial communities and their metabolites strongly influence sediment organic matter concentration, sediment pH, cycling, accumulation, and speciation of trace elements and essential nutrients in sediments [10,11,12]. For example, a greater abundance of the phyla Chloroflexi and WS3 have been reported in mangrove sediment than in adjacent seagrass sediment, highlighting the distinct microbial assemblages associated with mangrove environments [13]. Moreover, higher abundances of Chloroflexi and Proteobacteria, such as Planctomycetota, are dominant in mangrove sediments than in aquaculture pond sediments [14]. The functionality of mangrove ecosystems mostly depends on diverse microbial communities and benthic organisms, which are responsible for nutrient cycling within ecosystems to support mangrove seedlings in growing and building their forest [14,15,16].

Recently, mangrove restoration, reforestation, and afforestation have received increasing global attention due to their carbon sinks and storage capacity, positioning them as natural solutions for climate change mitigation and adaptation [17,18]. Recognizing their importance, the United Nations declared mangrove restoration as a key strategy for sustainable development goals for climate change mitigation and ecosystem protection [19]. Most countries in the world want to be carbon-neutral by the middle of this century under the Paris Climate Agreement deal [20]. As a result, several countries are taking initiatives and improving their existing plans for mangrove restoration or reforestation in coastal areas for carbon sequestration [20,21]. However, large-scale mangrove restoration or reforestation may induce complex ecological changes that influence multiple ecosystem processes [18]. These changes may include alterations in sediment properties, sediment pH, carbon sequestration, and trace element or nutrient cycling; changes in the bioavailability of toxic pollutants to benthic or aquatic species; and changes in soil microbial community diversity, soil microbial metabolic activities, and metabolites [22,23,24,25]. The soil microbial communities linked to mangrove ecosystems may differ due to the vegetation structure, location (seaward versus landward zones), seasonal variability, anthropogenic disturbance, restoration status, and pollution conditions (contaminated or not contaminated) [9,11,26,27].

For example, Ma et al. (2021) [13] demonstrated that sediment-borne trace elements significantly altered microbial diversity and community composition in contaminated mangrove sites than in less polluted or reference sites. Liu et al. (2019) [7] reported substantial spatial variation in microbial community composition among inner mangrove forests, mangrove edges, and seaward zones and further showed that differences in mangrove species also influence sediment microbial diversity and community structure [7]. However, these differences in microbial diversity and their interrelations with carbon storage and trace metal cycling have not been comprehensively synthesized. In particular, the mechanistic links between microbial diversity, carbon sequestration, and trace element accumulation and speciation remain fragmented across the existing literature. Therefore, a comprehensive synthesis is needed to integrate current knowledge, identify major research gaps, and develop a conceptual understanding of how microbial communities regulate carbon storage and trace element dynamics in natural and restored mangrove ecosystems.

Previous reviews on mangrove ecosystems have primarily focused on the following topics including the effects of climate change on nutrient dynamics [28], microbial research priorities in rehabilitating mangroves [29], biogeochemical processes in mangrove ecosystems [30], geographical and sectorial dimensions of coastal vegetated ecosystems [31], ecological interactions of microorganisms in mangrove ecosystems [8], microbial communities in blue carbon ecosystems [9], and the impact of ecological restoration on carbon sinks in coastal mangrove ecosystems [4]. However, no comprehensive review has systematically integrated the relationships among mangrove restoration, microbial diversity, carbon sequestration, trace metal accumulation, and speciation. Although numerous studies have investigated the understanding of mangrove microbial diversity and trace element cycling independently, the mechanistic links through which microbial communities simultaneously regulate trace element accumulation and speciation remains fragmented and poorly understood. This knowledge gap is particularly important because global mangrove restoration has been expanding due to climate change mitigation and coastal protection, but the influence of restoration on the transformation of carbon storage and trace elements in restored mangrove ecosystems has not been comprehensively synthesized [18].

A comprehensive synthesis of the current knowledge is needed to determine the possible differences in ecological functions between restored mangroves and natural mangroves, and how these alterations in ecological functions influence soil microbial community diversity, soil microbial metabolic activities, trace element accumulation, speciation, and nutrient cycling within coastal mangrove ecosystems. Such an integrated perspective will identify major research gaps, establish a conceptual framework linking microbial processes with carbon and trace element dynamics, and provide research priorities for future directions to support sustainable mangrove management, blue carbon conservation, and pollution mitigation.

The aims of this review are to provide a synthesis of current knowledge on the role of microorganisms in regulating carbon storage and trace element cycling in mangrove ecosystems. Specifically, this review aims to: (i) assess the global status and research trends in mangrove microbial studies; (ii) synthesize the influence of microbial mechanisms and functions on carbon storage in sediments, trace metal accumulation, and speciation in natural and mangrove sediments; (iii) identify current knowledge gaps and propose future research priorities to advance mechanistic understanding and support effective mangrove restoration and blue carbon management.

## 2. Research Status of Mangrove Microbial and Trace Element Interactions

**Figure 1:**
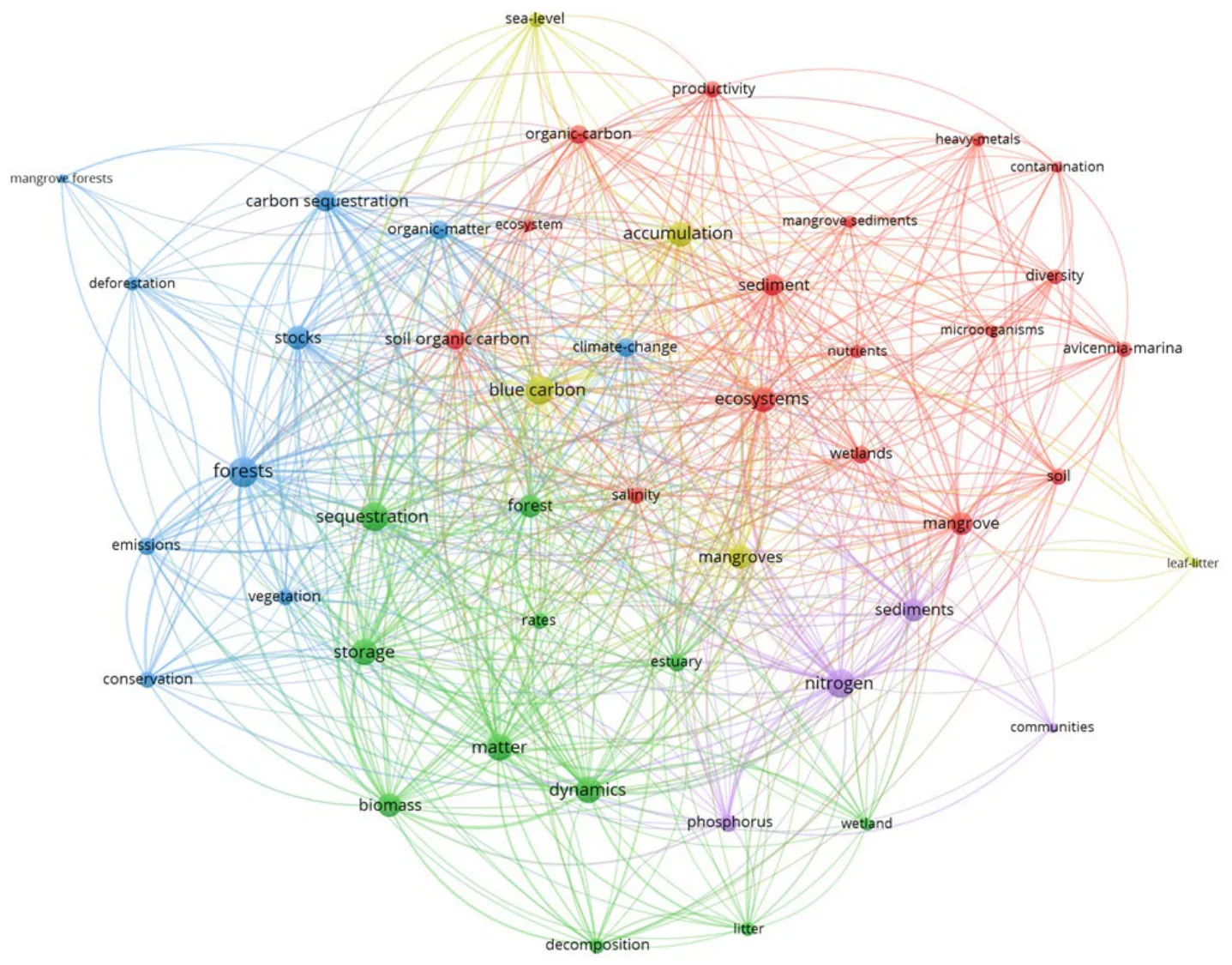
Bibliometric network visualization map-based co-occurrence of keyword network analysis.

To evaluate the current network analysis, research status, and global attention related to the influence of microbes on carbon storage, trace metal accumulation, and speciation in mangrove ecosystems, we conducted a comprehensive literature search using the “Web of Science (WoS)” database. The search was performed using combinations of the keywords “mangrove microbial carbon storage” and “mangrove microbial trace elements,” “mangrove sediments,” “microbial influence,” “carbon storage,” and “trace elements”. To improve the accuracy and credibility of the articles, the search was refined using article titles and author keywords that specifically addressed microbial processes in mangrove ecosystems. The selection and screening of publications followed the Preferred Reporting Items for Systematic Reviews and Meta-Analyses (PRISMA) guidelines [32]. Bibliometric network analysis was subsequently performed using VOSviewer version 1.6.20 to evaluate keyword co-occurrence patterns and quantify their relationships based on total link strength. The network co-occurrence analysis of network visualization is presented in Figure 1, providing an overview of the major research themes and their interconnections within the field.

**Figure 2:**
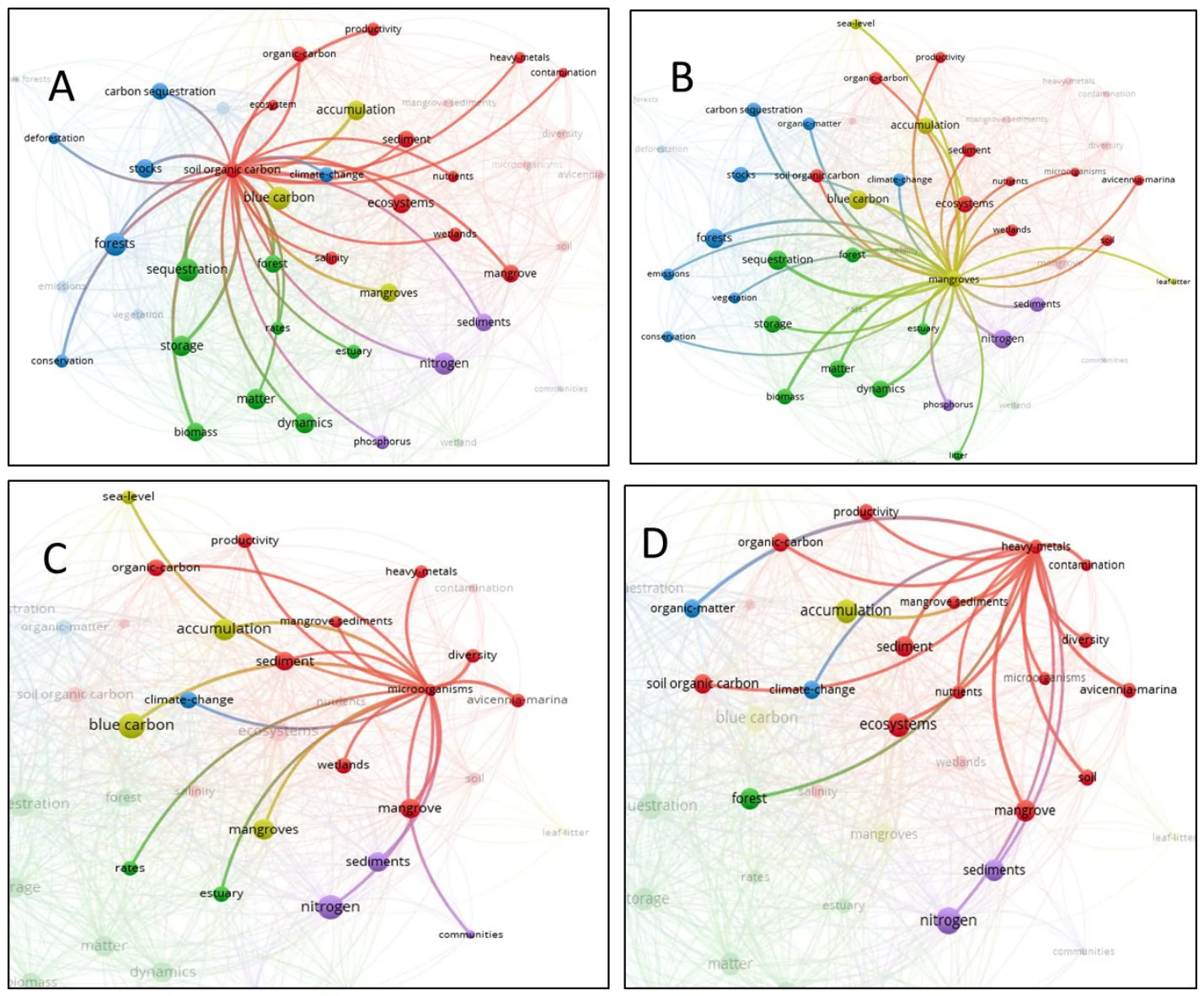
Bibliometric network visualization map of individual keywords ((**A**): soil organic carbon; (**B**): mangroves; (**C**): microorganisms; (**D**): heavy metals) based on co-occurrence network analysis.

In the network visualization, the label and circle are determined by the weight of the item, and the color indicates the cluster of similar items. The links between nodes indicate the co-occurrence relationships among keywords, with greater link strength reflecting stronger associations. The analysis identified seven different distinct keyword clusters, representing the major research themes within the field. The most frequently occurring and strongly connected keywords included forest, mangrove, blue carbon, nitrogen, accumulation, sequestration, storage, ecosystems, and dynamics. In addition, the occurrence frequencies and total link strengths of the individual keywords are summarized in Figure 2.

**Figure 3:**
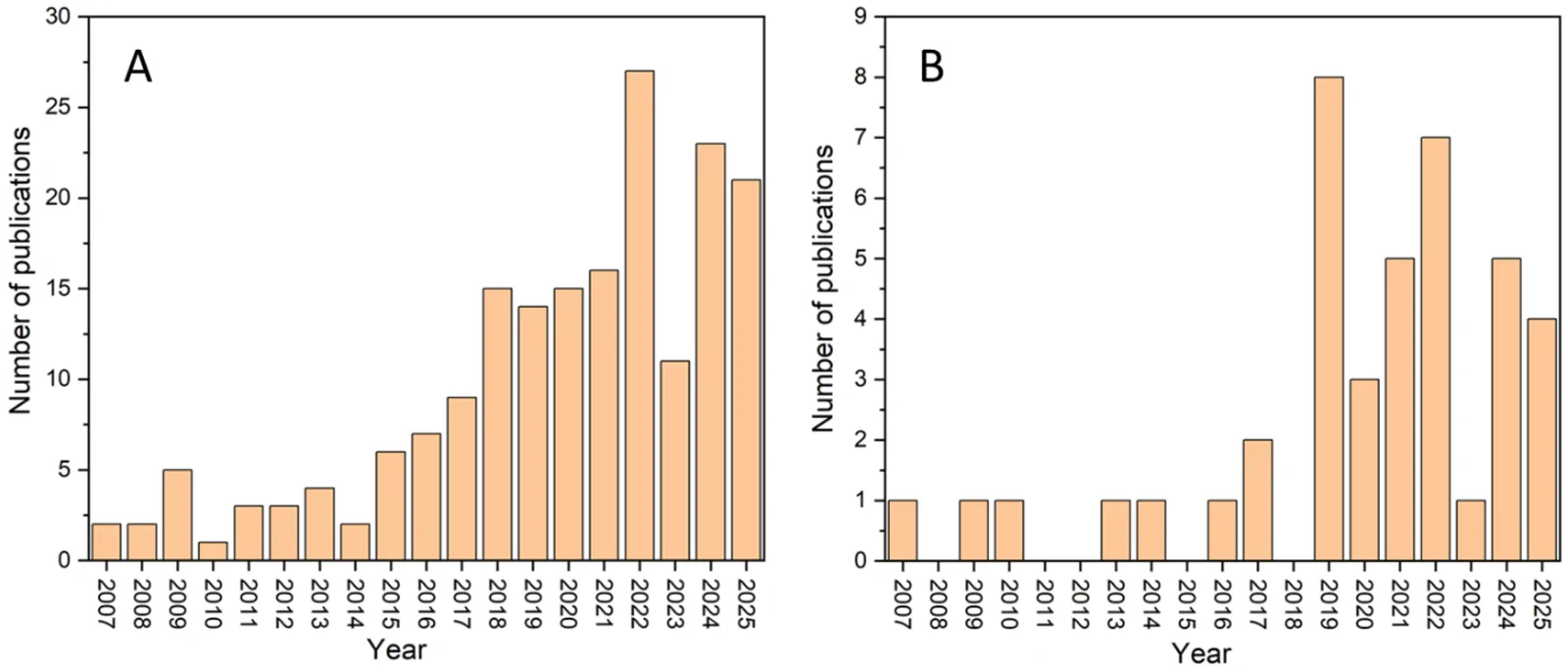
The number of research publications globally related to microbial carbon storage (**A**) and microbial trace metal interactions (**B**) in mangrove sediment between 2007 and 2025 (data source: Web of Science).

The keyword co-occurrence analysis revealed that mangroves and soil organic carbon were among the most frequently occurring and strongly connected research topics, whereas trace elements and microorganisms appeared less frequently and exhibited comparatively weaker link strengths. Nevertheless, the co-occurrence map showed that mangroves, soil organic carbon, microorganisms, and trace elements were grouped within the same cluster, indicating a close conceptual relationship among these research themes in the existing literature. To further evaluate research trends, we also investigated the current research status in this field. The bibliometric results show a substantial increase in studies investigating microbial regulation of carbon storage and microbial interactions with trace elements in mangrove ecosystems between 2007 and 2025 (Figure 3), reflecting the growing global interest in understanding the microbial mechanisms underlying mangrove biogeochemical processes.

**Figure 4:**
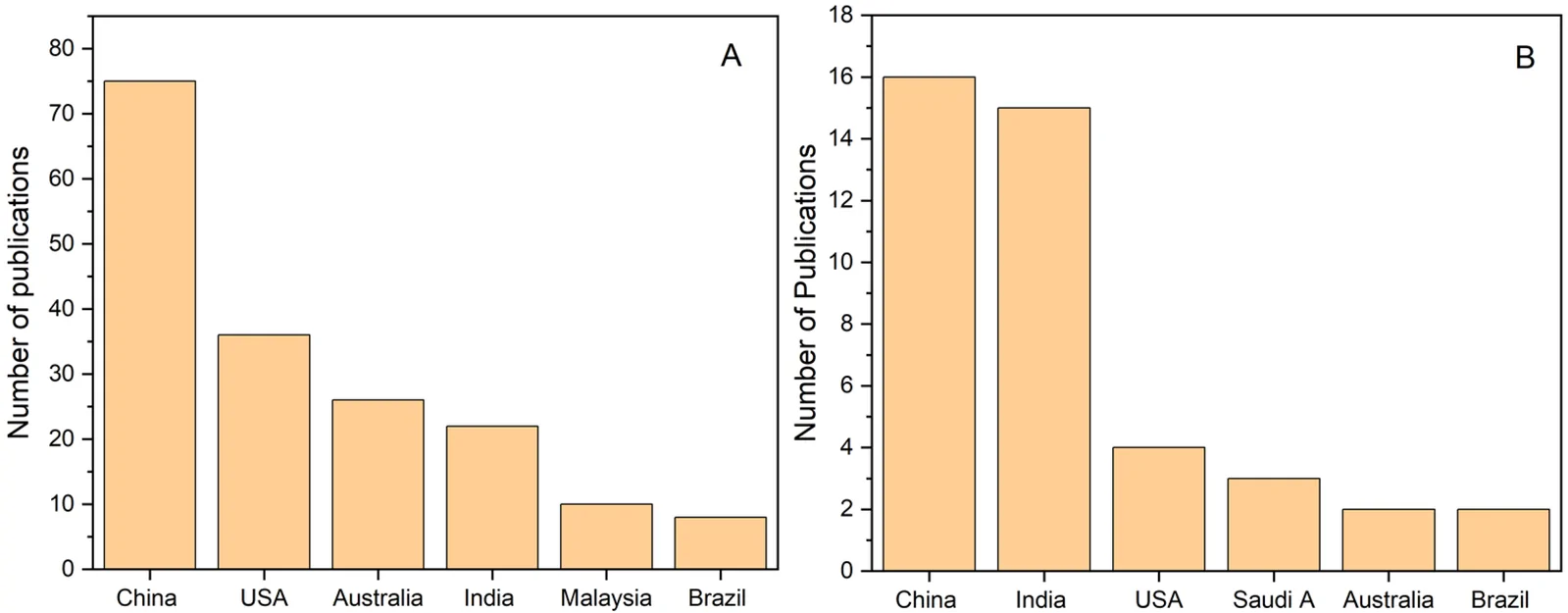
The top countries that published mangrove microbial carbon storage interactions (**A**) and mangrove microbial trace element interactions (**B**) in the world (data source: Web of Science).

The temporal analysis revealed a marked increase in publications on the role of microorganisms in mangrove sediment carbon storage after 2017, indicating growing scientific interest in the microbial mechanisms underlying blue carbon sequestration. In contrast, studies investigating microbial interactions with trace elements in mangrove ecosystems remain comparatively limited. Although research on microbial–trace element interactions has increased noticeably since 2018, the number of publications is still substantially lower than that of studies focusing on microbial regulation of carbon storage (Figure 3). We also analyzed the geographical distribution of research output to identify leading contributors related to microbial carbon storage interactions and microbial trace metal interaction research. According to the WoS database, China, the USA, and Australia produced more research articles about mangrove microbial storage interactions than other countries (Figure 4). Moreover, regarding mangrove microbial trace metal interactions, China, India, and the USA were the leading contributors (Figure 4).

## 3. Microbial Influence on Carbon Storage in Sediments

**Figure 5:**
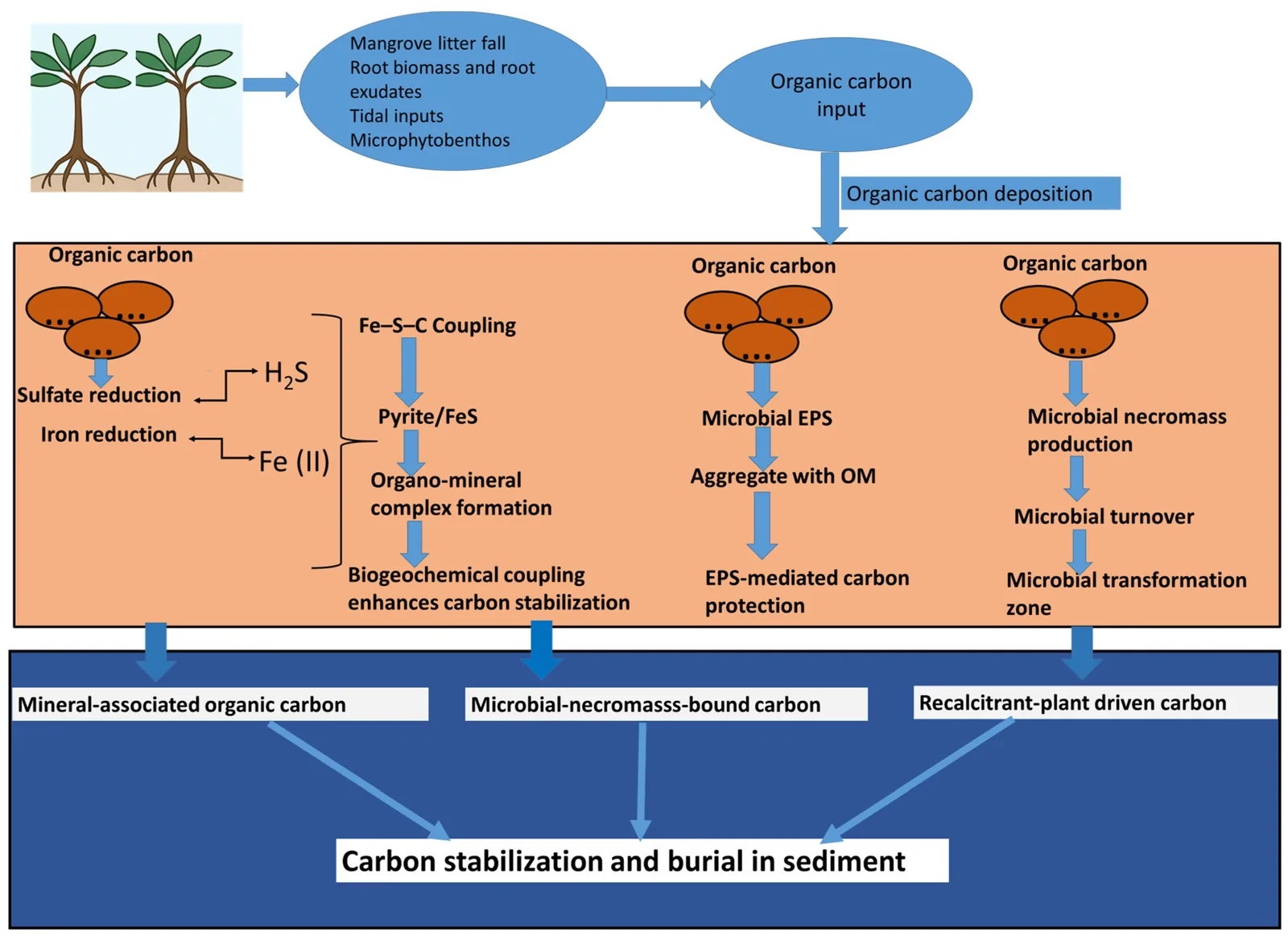
Conceptual diagram of the microbial regulation of carbon storage in mangrove sediments.

Microbial diversity is fundamental to the biogeochemical cycling and organic carbon storage in mangrove sediments through the heterotrophic metabolism of sediment microorganisms [33]. Mangrove sediments are anoxic and high in nutrient content and organic matter, which creates a special environment for microbial processes that supports diverse microbial communities and drives complex carbon cycling processes [34]. Mangrove sediments are hot spots for microbial abundance and diversity due to relatively high organic inputs, tidal influence, and sulfate-rich, fine-grained sediments [35]. These environmental conditions favor anaerobic microbial metabolism, which plays a central role in determining the fate of organic carbon by balancing its decomposition, transformation, stabilization, and long-term preservation [36]. Through a range of biogeochemical pathways, microorganisms regulate carbon storage by mediating the transformation, mineralization, and stabilization of organic matter under waterlogged and oxygen-limited conditions [37,38]. A conceptual framework illustrating the microbial regulation of carbon storage in mangrove sediments is presented in Figure 5.

Microorganisms are the primary agents of organic matter decomposition in mangrove sediments. However, the persistently waterlogged and oxygen-limited conditions characteristic of these environments constrain aerobic decomposition and favor anaerobic metabolic pathways, which generate less energy and proceed more slowly. As a result, the decomposition of organic matter is reduced, promoting greater carbon burial and long-term carbon preservation [3]. Sulfate-reducing bacteria (SRB) are considered as keystone species in mangrove sediments for carbon mineralization. SRB use sulfate as an electron acceptor, oxidize labile organic carbon, and produce hydrogen sulfide [39]. Hydrogen sulfide reacts with the iron content in sediment for the formation of pyrite and reduces the iron availability for organic matter oxidation. This microbial-driven iron–sulfur coupling enhances the stabilization and preservation of sediment organic matter, contributing to long-term carbon sequestration in mangrove ecosystems [38,40].

Microbes in mangrove sediments produce extracellular polymeric substances (EPSs) primarily composed of polysaccharides and proteins, and these compounds enhance the physical protection of organic matter through aggregation and mineral binding [41]. EPS-bound carbon is highly resistant to enzymatic degradation and less labile, while EPS act as a microbial shield for the protective microbial matrix that reduces the decomposition of sediment organic carbon [36,42]. Microbes and organo-mineral associations decompose organic matter into lower-molecular-weight compounds, and these compounds strongly bind with clay minerals and iron (Fe) or aluminum (Al) oxides to increase the stable carbon content in sediment [41,43]. In addition, microbial regulation of iron cycling further contributes to carbon stabilization. Iron-reducing microorganisms transform reactive Fe minerals, promoting the formation and persistence of Fe–organic matter (Fe–OM) complexes that enhance long-term carbon preservation under anoxic sediment conditions [38].

Multiple environmental drivers including redox potential, nutrients, restoration, land use changes, and pollutant inputs strongly influence microbial function or behavior in mangrove sediments, and those drivers also substantially influence carbon storage in mangrove sediments [44,45,46,47]. Among these factors, sediment redox conditions are particularly important because they regulate both microbial community structure and carbon fixation processes [47]. Under low redox (reducing) conditions, carbon-fixing microbial communities become more abundant, resulting in greater organic carbon in sediment. In contrast, higher redox (more oxidizing) conditions favor lower abundance of carbon-fixing microbial communities and enhanced rapid carbon mineralization [47]. Therefore, tide-bound nutrients significantly enhance microbial carbon metabolic functions and the microbial carbon cycle in sediments [44]. For example, in tidal-dominated Shenzhen Bay, mangrove sediments contained significantly higher dissolved inorganic nitrogen and phosphate than those in the less tidally influenced Daya Bay, resulting in distinct microbial community structures and metabolic functions [44]. These variations in nutrients favored the dominance of the family Desulfobacteraceae, whereas the family Anaerolineaceae was more abundant in less nutrient-rich sediments [44]. Furthermore, co-occurrence network analysis and gene-chip analysis revealed that tidal-dominated sediments possessed more complex interaction networks and carbon-related functional genes than less tidally influenced sediments, resulting in greater carbon storage in tidal-dominated nutrient-rich areas [44].

Land-use conversion, and artificial and natural mangrove restoration have substantial influence on the soil aggregate stability and microbial networks in mangrove sediments [46]. For example, the conversion of aquaculture ponds to mangrove forest has been shown to enhance soil stability and carbon sequestration through increased microbial diversity [48]. However, natural and artificial mangrove restoration results in variation in microbial symbiosis; natural restoration increases Desulfobacterota and Basidiomycota microbial communities, which results in greater carbon sequestration or storage in natural mangrove sediment than in artificially restored mangrove sediment [48]. In contrast, environmental pollutants including trace elements and microplastics can disrupt the normal growth of microbial diversity, reducing microbial-mediated carbon sequestration and decreasing sediment carbon storage by approximately 8–12% [45]. Collectively, these findings demonstrate that microbial communities are central regulators of carbon storage in mangrove sediments, while environmental drivers such as restoration strategy, land-use change, and pollution shape microbial activities and ultimately determine the efficiency of blue carbon sequestration.

## 4. Microbial Influence on Trace Element Accumulation and Speciation

**Figure 6:**
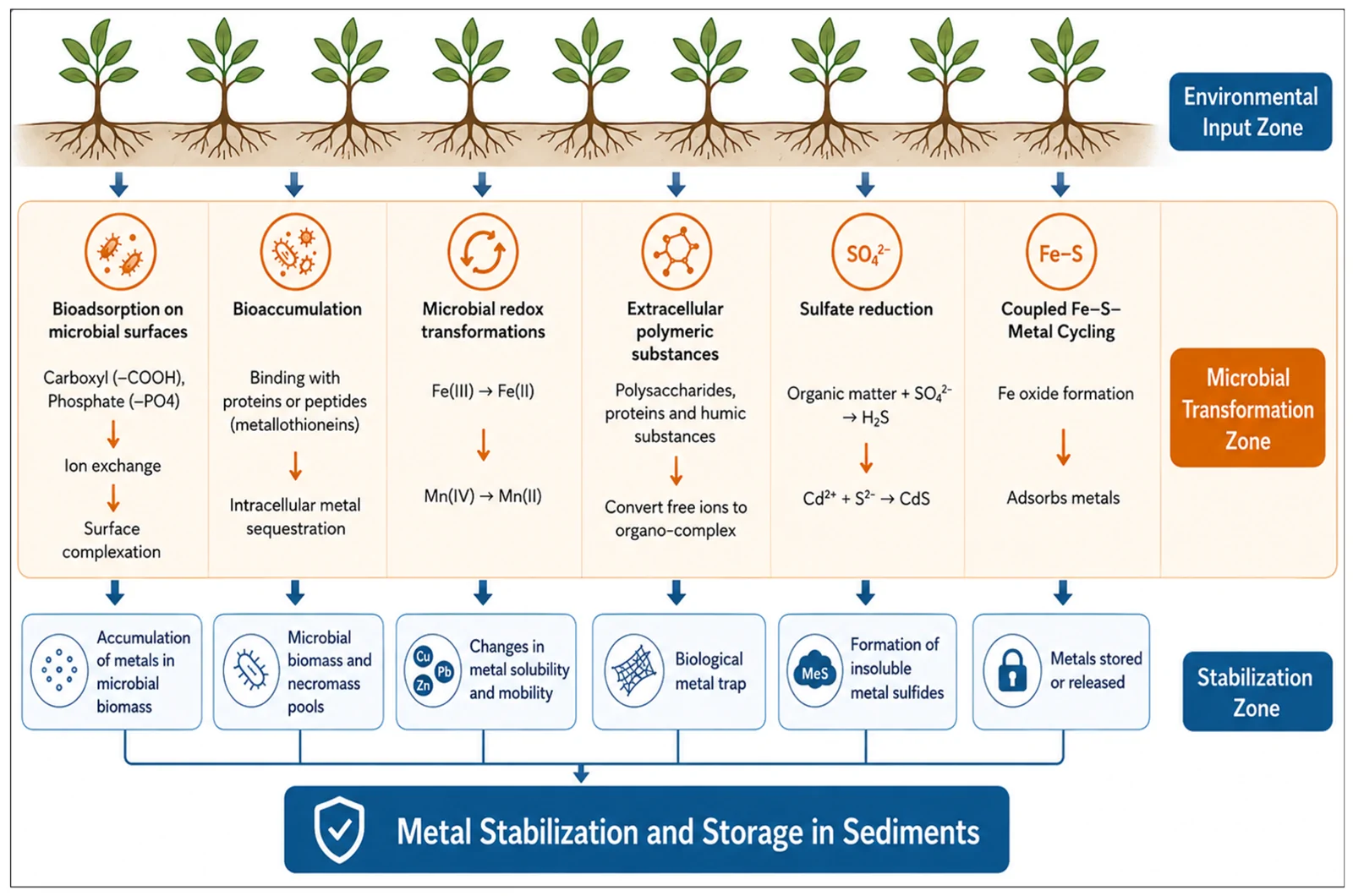
Conceptual diagram illustrating the microbial regulation of trace element accumulation and speciation in mangrove sediments.

Microorganisms play a central role in regulating trace element accumulation, speciation, and mobility in mangrove sediments. Therefore, microbial diversity in mangrove sediments influences different processes, such as redox reactions, organic complexation, and mineral transformation, and those processes determine element speciation, mobility, bioavailability, and risks in sediments [8,49,50]. A conceptual framework illustrating the microbial regulation and mechanism of trace element accumulation and speciation is presented in Figure 6.

The accumulation, speciation, and mobility of trace elements in mangrove sediments are largely regulated by microbial activity through a range of interconnected biochemical and geochemical processes [10]. These processes interact with trace elements to determine whether trace elements remain mobile and bioavailable or become immobilized and sequestered within the sediment matrix [51]. Microbial communities regulate trace element dynamics through several mechanisms, including redox transformations, bioaccumulation and biosorption by microbes, EPS-mediated binding and aggregation, biomineralization, and sulfide precipitation [12,52,53,54]. These processes interact with the iron and sulfur cycles to control metal immobilization, mineral associations, and long-term accumulation in sediments. Conversely, environmental disturbances, such as changes in redox conditions, salinity, or sediment chemistry, can destabilize these associations and enhance trace element remobilization and bioavailability [12,52].

Microorganisms regulate trace element speciation or alter their chemical forms through redox reactions, enzymatic transformation, and metabolic byproducts in mangrove sediments [22]. Through these processes, microbial metabolism modifies the oxidation states of trace elements. For example, iron-reducing bacteria reduce ferric iron [Fe(III)] to ferrous iron [Fe(II)], manganese-reducing bacteria, arsenate-reducing bacteria, and microbial methylation [15]. These transformed or reduced forms of elements are more favorable and mobile in aquatic environments and may pose environmental risks [55,56]. In addition to redox transformations, microorganisms in mangrove sediments can directly take up trace elements in their cells through passive adsorption, intracellular uptake, and binding with intracellular proteins. Following microbial cell death, the accumulated elements may become incorporated into microbial biomass and necromass, contributing to sediment-bound trace element pools and influencing their long-term cycling and retention within mangrove ecosystems [10].

In addition to intracellular uptake, microorganisms immobilize trace elements through biosorption on their cell surfaces and within extracellular polymeric substances (EPS). Microbial EPS are primarily composed of polysaccharides, proteins, lipids, and humic-like substances [57]. These substances are strongly attached to trace elements through electrostatic attractions, complexation, ion exchange, and surface precipitation, resulting in reduced mobility of trace elements in sediments and increased element retention in sediments [58]. Because of their capacity to immobilize metals, EPS have been described as a biological metal trap.

Sulfate-reducing bacteria are important in regulating trace element sequestration in mangrove sediments [59]. During anaerobic respiration, SRB utilize sulfate as a terminal electron acceptor and generate hydrogen sulfide as a metabolic by-product. The produced sulfide subsequently reacts with dissolved trace metals to form insoluble metal sulfides, thereby immobilizing trace elements and promoting their long-term sequestration within the sediment matrix [54,59,60]. In contrast, microbial degradation of organic matter can remobilize trace elements previously associated with organic matter by enhancing mineralization, dissolution of element—organic matter complexes, and reduction of Fe/Mn oxides [60]. In this process, trace elements enhance mobility and remobilization during environmental change.

The mangrove rhizosphere further regulates microbial–trace element interactions by modifying sediment physicochemical conditions [40]. Mangrove roots can release oxygen into sediments, create localized redox gradients and provide a substrate for organic matter binding [40,52]. These processes can increase Fe/Mn oxide formation, increase the adsorption of trace elements, and stabilize elements around mangrove rhizosphere sediments [61]. Mangrove restoration or reforestation can increase trace element accumulation in mangrove sediments compared with unvegetated mudflats or degraded coastal areas [22,23]. Therefore, mangrove restoration can increase microbial diversity, which affects the microbial decomposition of mangrove leaves, roots, and bark in sediments, resulting in an increase in the organic carbon content in sediments.

In the Futian Mangrove Nature Reserve, the methylation of mercury has been shown to be strongly influenced by microbial diversity [12]. Mangrove ecosystems have higher organic carbon, which promotes growth and modifies Hg methylators as a result, increasing methyl mercury in mangrove sediments [12]. Sulfate-reducing bacteria such as Desulfococcus and Desulfosarcina, together with syntrophic bacteria including Syntrophus, are closely associated with Hg methylation through anaerobic metabolic pathways, including the acetyl-CoA pathway, under anoxic sediment conditions [12]. These findings highlight the important role of microbial communities in controlling mercury transformation and its potential ecological risks in mangrove ecosystems.

EPS are widely observed in microbial cells or the intercellular space of microbial aggregates in both restored and natural mangrove sediments [62]. EPS have different metal-chelating functional groups, such as hydroxyl, carboxyl, and amine groups, which effectively chelate trace elements and promote their adsorption, immobilization, and long-term retention in sediments [63]. Compared with unvegetated mudflats, restored mangrove ecosystems generally exhibit greater microbial diversity and enhanced EPS production, providing additional binding sites for trace elements and strengthening microbial-mediated trace element retention in sediments [62,63].

A study conducted in restored mangrove in Fucheng, China, reported greater EPS production and microbial diversity than non-restored area sediment [10]. Therefore, restored mangrove sediments exhibited greater accumulation of Cr, Cu, Ni, Pb, and Zn than in non-restored areas [10]. This study also revealed that higher accumulation of trace elements in mangrove sediments is positively correlated with EPS production and microbial diversity [10]. However, EPS and microbial diversity are negatively correlated with trace element bioavailability or mobility. These findings suggest that microbial communities and EPS promote the immobilization and long-term sequestration of trace elements while reducing their mobility and potential ecological risks in mangrove sediments [10]. Consequently, the enhanced retention of trace elements in restored mangrove sediments may reduce the transfer of contaminants to adjacent coastal ecosystems [22].

Natural mangroves generally support more diverse and functionally mature microbial communities than restored forests, resulting in more efficient trace element stabilization [61]. In particular, sulfate-reducing bacteria are typically more active in natural mangrove sediments, promoting the precipitation of trace elements as insoluble metal sulfides. In contrast, recently restored mangroves, especially during the early stages of ecosystem development, often exhibit lower sulfate-reducing activity, causing a larger proportion of trace elements to remain in more labile exchangeable or carbonate-bound fractions [61]. As restored mangroves mature, microbial diversity and metabolic activity are expected to increase, progressively enhancing trace element immobilization and long-term sediment stabilization [61].

Overall, in mangrove ecosystems, microbes act as burial agents or accumulate trace elements in sediments and influence trace element mobility in mangrove sediments. Microbial diversity and its role are crucial to understanding element dynamics, accumulation, speciation, risks, and remediation strategies in this critical ecosystem.

## 5. Research Gaps, Future Research Directions, and Recommendations

**Figure 7:**
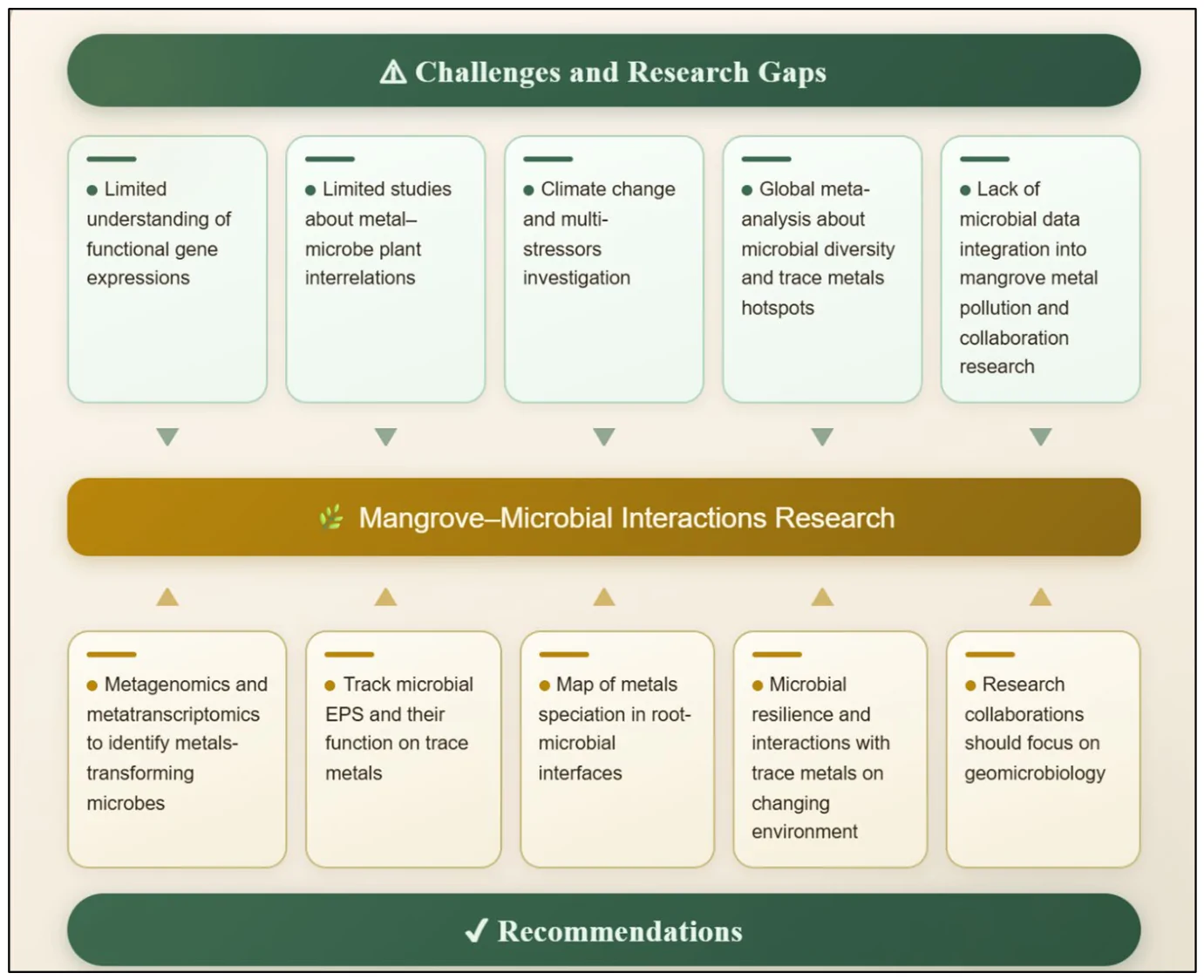
Graphical illustration of research challenges, research gaps, and recommendations for mangrove–microbial trace element interaction research.

Mangrove–microbial interactions are fundamental to the regulation of carbon sequestration and trace element accumulation, speciation, and mobility, with important implications for ecosystem conservation, pollution remediation, risk management, and climate change adaptation and mitigation. Mangroves are critical for carbon sinks and the filtering of trace elements, but the influence of microbes on these functions in restored and natural mangrove systems is still understudied (Figure 2). The major research challenges, research gaps, and future research priorities for mangrove–microbial interactions are summarized in Figure 7.

Most studies investigating microbial–trace element interactions in mangrove ecosystems have focused on diversity changes in mangrove sediments compared with those in mudflats, comparisons within mangrove sites, variations in inundation, polluted or microplastic-contaminated areas, and restored and afforested mangrove areas [10,15,64]. Therefore, different genetic tools have been used to identify microbial abundance and diversity, but the dynamics of microbial consortia under different trace element pollution or stress conditions are still understudied. The understanding of functional gene expression for sulfate reduction (*dsrB*) or trace element detoxification (*merA* for Hg detoxification) is very limited [65]. To address these knowledge gaps, future research should integrate metagenomic and metatranscriptomic approaches to identify functional microbial taxa and characterize the genes and metabolic pathways responsible for trace element transformation [65]. In addition, single-cell genomic, proteomic, and metabolomic approaches should be employed to investigate the metabolic functions of uncultured microorganisms, elucidate microbial responses to trace element stress, and clarify the role of extracellular polymeric substances (EPS) in trace element immobilization and biogeochemical cycling [66,67].

Microbial processes in mangrove sediments strongly influence trace element accumulation, speciation, and mobility, thereby regulating trace element uptake by mangrove plants. Consequently, interactions among plant roots, rhizosphere microorganisms, and trace elements are fundamental to trace element stabilization, phytoextraction, and phytoremediation in mangrove ecosystems [33]. However, the mechanistic relationships among roots, microorganisms, and trace elements at the microscale remain poorly understood, limiting our ability to predict and optimize contaminant stabilization and remediation processes [61]. To address this gap, future studies should employ synchrotron-based imaging to quantify maps of trace element speciation at root—microbial interfaces [68]. In mangrove ecosystems, tidal fluctuations, salinity gradients, and anoxic conditions are very common and influence microbial trace element cycling. In addition, emerging contaminants such as microplastics can alter microbial community structure and potentially influence trace element adsorption, transformation, and transport. However, the interactions among microplastics, microbial communities, and trace elements remain poorly understood [69]. We therefore recommend controlled simulated or artificial mesocosm experiments with fluctuations in tidal inundation and changes in pH and salinity to understand microbial resilience and interactions with trace elements in a changing environment [70]. Resilience and its influence on trace element cycling under changing environmental conditions are of interest [70]. Future research should also prioritize integrated investigations of microplastic–microbe–trace element interactions to improve our understanding of contaminant dynamics and support the sustainable management and restoration of mangrove ecosystems [71].

Microbial–trace element interactions in mangrove ecosystems are strongly influenced by regional differences including climatic setting, and tropical and temperate mangrove ecosystems [72]. Future research should therefore prioritize global meta-analyses and comparative studies on trace element and microbial datasets in different biogeographical regions.

From a management perspective, microbial processes remain underrepresented in mangrove pollution assessment frameworks and conservation policies [73]. Likewise, information on microbial–trace element interactions is rarely incorporated into citizen science initiatives or public outreach programs [74]. Future research should integrate microbial-based indicators and microbial-health metrics into mangrove conservation strategies and policy frameworks [75].

Advancing our understanding on mangrove microbial trace element interactions in mangrove ecosystems requires stronger interdisciplinary collaboration among researchers from complementary scientific disciplines; those are essential for identifying research gaps and finding a new way of setting research priorities [76]. Future collaborative studies should focus on geomicrobiology, such as microbial trace element and mineral interrelations; biogeochemistry, such as tracing microbial and element pathways; ecotoxicology, such as risk to benthic fauna; and data science for modeling or predicting microbial mediated element fluxes [77,78,79]. Current research has primarily emphasized the role of complete-oxidizing sulfate-reducing bacteria, particularly Desulfococcus and Desulfosarcina, in regulating trace element transformations [12]. However, the contributions of incomplete-oxidizing sulfate-reducing microorganisms and other functional microbial groups remain poorly understood. Future studies should therefore investigate the diversity, metabolic pathways, and ecological functions of these less-studied microbial taxa to develop a more comprehensive understanding of microbial regulation of trace element cycling in mangrove ecosystems [12].

Although recent studies have increasingly investigated microbial production of extracellular polymeric substances (EPS) in mangrove sediments, the mechanisms governing EPS synthesis and its role in trace element sequestration remain poorly understood. Future research should prioritize bacterial isolation, EPS synthesis, and trace element sequestration [10]. The mangrove rhizosphere supports diverse soil microorganisms including both beneficial and pathogenic microbes. While beneficial microbes have been extensively studied because of their roles in nutrient cycling and ecosystem functioning, comparatively little is known about the ecological roles of pathogenic microorganisms and their responses to environmental stress. Future studies should identify indicator species for environmental stress and environmental shifts as well as their application in sustainability [54].

Microbial abundance and community composition are closely associated with the inorganic and organic pollution status of sediments. Accordingly, future research should focus on developing microbial bioindicator frameworks for monitoring pollution, ecosystem health, and restoration success in mangrove ecosystems [59]. Overall, future investigations should focus on multi-omics approaches, environmentally linked dynamics, mesocosm experiments with multiple stress conditions, and interdisciplinary collaborations between microbial processes and trace element management. Establishing multidisciplinary research frameworks together with regional and global data-sharing platforms will be essential for identifying key knowledge gaps, improving predictive capabilities, and supporting evidence-based conservation, restoration, and sustainable management of mangrove ecosystems.

## 6. Conclusions

This review synthesizes current knowledge on the roles of microorganisms in regulating carbon storage and trace element cycling in natural and restored mangrove ecosystems, while evaluating global research trends and identifying key knowledge gaps. In terms of research status, trace element and microbial interactions in mangrove ecosystems are still less explored than are carbon microbial storage interactions. However, after 2018, microbial trace metal interaction research in mangrove sediments increased considerably compared with that in previous years. The available evidence demonstrates that microbial communities are central regulators of sediment carbon sequestration through interconnected processes, including sulfate reduction, biogeochemical iron–sulfur coupling, extracellular polymeric substance (EPS)-mediated stabilization, microbial necromass formation, and other microbial transformation pathways. Therefore, microbial communities are also influenced by trace element accumulation and speciation in mangrove sediments through redox reactions, adsorption, EPS binding, and intracellular metal sequestration. Collectively, these findings indicate that microbial communities function as a critical biogeochemical bridge linking carbon storage with trace element dynamics in mangrove sediments. Despite these advances, future research directions should integrate metagenomic or metatranscriptomic analysis to identify metal-transforming microbes, tracking EPS functions for trace elements, mapping trace element speciation at root—microbial interfaces, assessing microbial resilience under multiple environmental stressors, and promoting interdisciplinary and international collaborations.

## Data Availability

No new data were created or analyzed in this study. Data sharing is not applicable to this article.
